# The Burden of Cystic Echinococcosis in Kenya: A Review Article

**Published:** 2019

**Authors:** Eshrat Beigom KIA, Francan Felix OUMA, Chrispinus Siteti MULAMBALAH, Patrick Kirsteen OKOTH

**Affiliations:** 1. Department of Medical Parasitology and Mycology, School of Public Health, Tehran University of Medical Sciences, Tehran, Iran; 2. Department of Medical Microbiology and Parasitology, School of Medicine, Moi University, Eldoret, Kenya; 3. Department of Biological Sciences, Masinde Muliro University of Science and Technology, Kakamega, Kenya

**Keywords:** Cystic echinococcosis, Prevalence, Diagnosis, Risk-factors, Kenya

## Abstract

**Background:**

Cystic echinococcosis (CE), caused by the larval stage of *Echinococcus granulosus* sensu lato (s.l), is a zoonotic parasitic disease with a worldwide distribution. Kenya is one of the high endemic countries of CE with the endemic areas in the country being under immense occupation of traditional pastoralists. Turkana area in Kenya, has in the past recorded the highest prevalence of CE in the world.

**Methods:**

The keywords cystic echinococcosis; Prevalence; Diagnosis; Risk-factors; Kenya were searched on google scholar and PubMed and the important literature materials retrieved for further analysis.

**Results:**

The most notable infection risk factor for this disease in the country is the close association between man, dogs, and livestock. Successful control of CE in Kenya requires application of innovative interventions achieved after the review of the disease situation in the country. With the emergence and advent of new diagnostic techniques, CE organ-specific infections and transmission pattern in Kenya differ from what is commonly reported in literature.

**Conclusion:**

A better understanding of CE prevalence of different hosts, its transmission pattern and the pathogenicity might make it possible to set up more effective control programs in future.

## Introduction

Cystic echinococcosis (CE) is a neglected but re-emerging zoonotic parasitic disease caused by the larval stage of tapeworm of species belonging to *Echinococcus granulosus* sensu lato (s.l) of the genus *Echinococcus* (1–3). The disease has worldwide distribution especially among rural traditional pastoralists where dogs are accessible to offal from slaughtered animals. In Kenya, the high prevalence of CE has been reported in two transhumant pastoralist communities, the Turkana and the Maasai where it is of high public health and veterinary concern (4, 5).

*E. granulosus* s.l complex has been subdivided into five cryptic species based on morphological differences, host specificity, and through molecular characterization based on mitochondrial DNA (mtDNA) and nuclear genomes. These sub-species include: *E. granulosus* sensu stricto (s.s; constituting of genotype G1 and genotype G3), *E. felidis* (formely lion strain), *E. equinus* (horse strain, G4), *E. ortleppi* (cattle strain, G5) and *E. canadensis* (camel strain, G6; pig strain, G7; and cervid strains, G8 and G10). *E. granulosus* s.s is the chief causative agent of CE in humans; the sheep is ideally the intermediate host (3, 6, 7).

Cystic echinococcosis is of public health and veterinary concern in the endemic areas. Infection to human occurs through the ingestion of the parasite eggs in food or water contaminated with dog feces or from direct contact with dogs (2). In human CE may cause acute disease and severe complications including life-threatening anaphylactic shock that can result from the rupture of cyst. The morbidity of the disease in human depends on the size and the number of cysts, the infected organ and the level of the immune response of the infected individuals. The occurrence of post-surgery death rates and relapses of CE in human are estimated to be 2.2% and 6.5%, respectively (2, 8). In livestock CE infection results in massive production and economic losses to both beef and dairy industries in endemic areas especially where animal husbandry is practiced and where dogs are accessible to offal of livestock from slaughterhouses. CE results in the loss of 1–3 million disability-adjusted life years (DALYs) every year and US$ 3 billion is used annually for treating medical cases and compensating losses in the livestock industry (1, 2).

Kenya is one of the highest CE endemic countries in the world with Turkana County in the northwestern part of the country has in the past recorded the highest global prevalence of CE (7). Most CE prevalence data in Kenya originate from the high endemic areas of Turkana (in the Northwest of Kenya), and Maasailand (which include Kajiado and Narok counties). These areas are in vast occupation of traditional transhumant pastoralists (9).

The aim of this narrative review article was to highlight the current prevalence, distribution, and risk factors of CE in Kenya, the role of both domestic and wild animals in transmission and to underscore the current intervention strategies used to curb the transmission and factors that obstruct these strategies. The gathered information will be available for the policy makers for more innovative interventional measures.

## Methods

The keywords were searched on the advanced search of PubMed and Google Scholar and Embase databases using the following search criteria (Echinococcosis [Title]) OR (Hydatid disease [Title]) OR (Cystic Echinococcosis [Title]) OR (Echinococccus [Title]) OR (E. granulosus [Title]) AND Kenya. For a comparison of changes in trend and pattern of the CE overtime, the search was made flexible enough to include the studies done in the 1980s. Any duplication that resulted from the three databases was removed. A manual searched was made to find other bibliographies that were not available in the databases

## Results and Discussion

### Species of Echinococcus and cystic echinococcosis in Kenya

The genotyping of *E. granulosus* based on Polymerase Chain Reaction-Restriction Length Polymorphism (PCR-RFLP) targeting the NADH dehydrogenase subunit 1 (nad 1) gene (6, 8) has revealed the existence of four species of *Echinococcus* in Kenya: *E. granulosus* s.s which is the most causative agent of CE in human in Kenya has been reported in livestock (cattle, sheep and goats) and wild herbivores in the conservation areas from various parts of the country (7, 5). *E. canadensis* (camel strain G6) has been reported in camels and goats in Turkana and Maasailand and in human patients from Turkana area (5, 7). *E. ortleppi* (cattle strain, G5) has been reported irregularly in cattle and pigs in southern and western Kenya (4, 5); *E. felidis* DNA has been detected in the fecal samples of wild carnivores including lion from the conservation areas of southern and central Kenya (8, 5, 10–12).

### Distribution and prevalence of cystic echinococcosis in Kenya

**Fig. 1:**
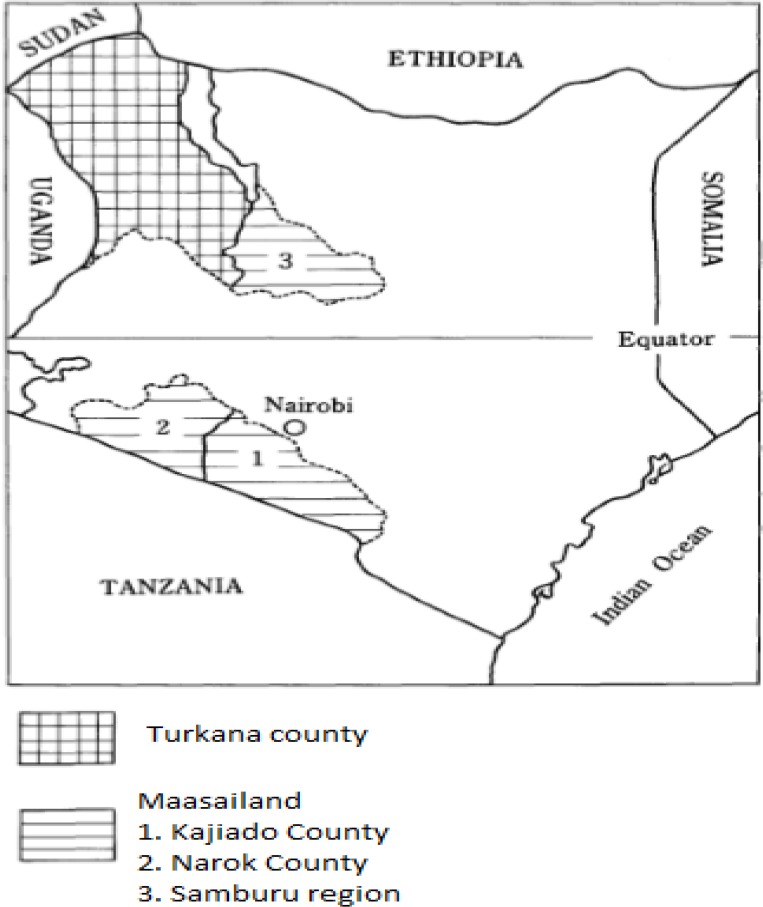
Cystic echinococcosis endemic areas in Kenya (4)

**Fig. 2:**
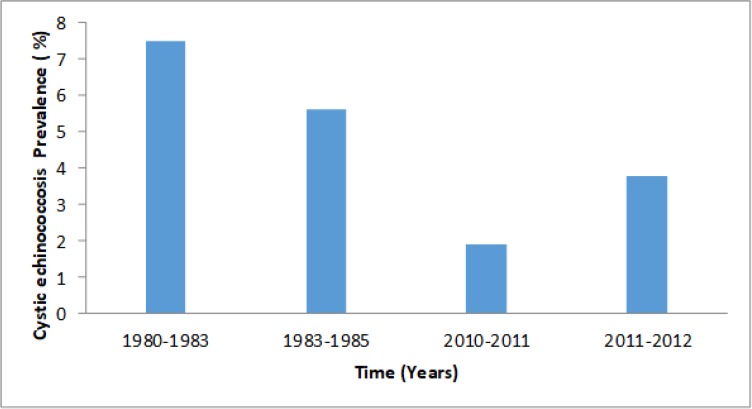
The prevalence of cystic echinococcosis in Turkana area between 1980s and 2012 (11)

Most of the CE prevalence survey data from livestock intermediate hosts have been generated mainly from the slaughter houses thus may be difficult to determine the origin of the livestock (4). Despite the predisposing factors evident in various parts of the country, most of the surveillance on humans have been emphasized from transhumant pastoralists colonized areas of Turkana, Kajiado, Narok and Samburu counties of Kenya (Fig. 1) (4, 5, 13, 14). Turkana County carries the highest prevalence in the country which was 7.5% in early 1980s but has declined to about 3.8% by 2012 (Fig. 2) (11). This reduction in prevalence in Turkana area is as a result of the control programs spearheaded by The AMREF from 1983 to 2012 (9, 11).

In Turkana area, early reports from humans indicated high prevalence of CE in ages between 20 and 40 yr (4) and the higher prevalence in females than males with the male to female ratio being 1:2. However high prevalence was reported in the age groups of 0–5 and 6–11 (9), these trends could not be explained. Solomon et al. in their cross-sectional screening survey reported a shift in prevalence to older age groups and a reducing prevalence in females with the females of childbearing age having the highest prevalence (4, 11). The prevalence patterns in other areas of Kenya are not well documented.

In domestic animals from various part of Kenya, the cattle are the most infected domestic intermediate host while camels carry the highest cyst burden among the domestic intermediate hosts (Table 1) (15). The sheep contributes highly in transmission in various parts of Kenya as they contain the cysts with the highest fertility rate (7).

**Table 1:** The prevalence of hydatidosis in domestic animals from selected regions of Kenya

***Region***	***Cattle***	***Sheep***	***Goats***	***Camels***	***Pig***	***Year***	***References***
Turkana	19.4%	3.6%	4.5%	61.4%	-	2002	(15)
Maasailand	25.8%	16.5%	10.8%	-	-	2012	(5)
Kisumu	4.0%	4.5%	2.0%	-	0.05%	2015	(4)
Isiolo	6.0%	1.0%	1.0%	25.3%	-	2015	(4)
Meru	1.92%	4.62%	0.37%	6.94%	-	2014	(9)

### Cystic echinococcosis specific organ involvement

The biggest cyst reported in Kenya was isolated from Kakuma Mission Hospital (KMH), Turkana, in 2013 from which 26 liters of fluid was drained (4).

**Fig. 3:**
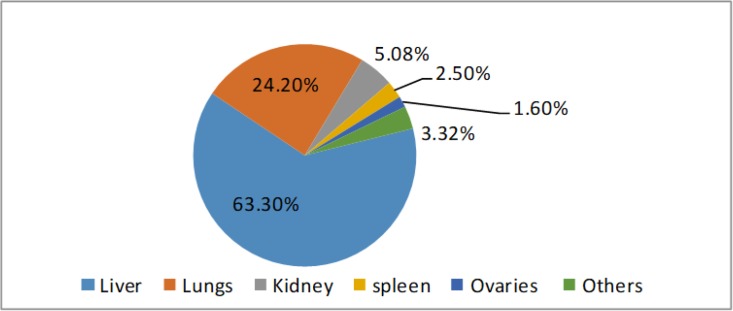
The intermediate host organ preference of hydatid cyst

Besides the CE common organs infections, there have been case reports of CE from humans in the heart, bladder, spine and the skin (Fig. 3, Table 2) (16–18). Bone (osseous) hydatidosis in human is also becoming common (19).

**Table 2:** Cystic echinococcosis human case reports from Kenya (except lungs and liver)

***Organ***	***Age (yr)***	***Gender***	***County***	***References***
Bladder	31	Male	Kiambu	17
Heart	30	Male	Turkana	16
Spine	23	Male	Turkana	18
Eye orbit 97 cases)	-	Males and Females	Various parts of Kenya	20

### Epidemiological significance of various hosts and risk factors in cystic echinococcosis transmission in Kenya

Several hosts both domestic and wild have a significant influence on CE transmission and epidemiology in Kenya. These animals can maintain the disease in a locality and/or enhance the spread to neighboring areas.

The domestic dog (*Canis familiaris*) is the main definitive host for CE in Kenya (4). Wild carnivores such as jackals, hyena, lion, cheetah and leopard are also definitive hosts for this parasite (14). Definitive hosts get infected by feeding on offal of both domestic and wild herbivores bearing the cysts (12, 20, 21).

The intermediate hosts of CE in Kenya include domestic and wild herbivores, omnivores ungulates and human. The intermediate hosts get infected by ingesting the parasite’s egg in pasture, water, or food contaminated with the infected definitive host’s feces (4). Human is said to be an accidental intermediate host (2). Wild herbivores such as wildebeest (*Connochaetes taurinus*) and single topi (*Damaliscus korrigum*) infection with hydatid cyst have been reported in southern Kenya. No wild herbivore infection has been reported in Turkana (4, 5, 13, 14)

### Risk factors for cystic echinococcosis in Kenya

Unhygienic living conditions, insufficient clean drinking and domestic water, insufficient public education activities as well as the close contact between human and dogs among the pastoralists are seen to foster the CE infection. Sharing of water holes among people, livestock, and dogs in dry endemic areas also play a role in CE infection. The high cyst burden in camels especially Turkana area could be as a result of drinking a lot of water from wells contaminated with dog feces bearing the *E. granulosus* eggs (22). Dogs have been reported to be used by women as baby nurse to clean babies of their defecation and vomiting as well as cleaning plates and pots in Turkana areas during the prolonged periods of drought and this contact fosters transmission (4, 22). Contaminated dust borne infections have also been suspected in dry and drought-hit areas in Kenya such as Turkana (12, 23).

Turkana, one of the highest endemic area in the country, borders three high CE endemic countries (Uganda, South Sudan and Ethiopia). Constant migration of animals from these neighboring countries and cattle rustling may also lead to the maintenance of the transmission cycle (15, 24, 25). A study conducted on ultrasonography on goats from South Sudan showed high prevalence on these goats compared to the goats in Kenya and most of these goats are being bought by the Kenyan slaughter houses.

### Diagnostic, treatment and control methods for cystic echinococcosis in Kenya

The most commonly used diagnostic methods of CE in human include ultrasonography (US), computerized tomography (CT) and magnetic resonance imaging (MRI). US a noninvasive method which provides immediate visual results can be used in the field which is unlikely with other diagnostic methods, however, the US is marred with challenges in detecting CE infections in bones and lungs due to its inability to penetrate through these organs. Serological methods are also being explored though it has low sensitivity and specificity and cannot give a lot of information about the cyst with regard to size, location and cyst stage (11).

In domestic intermediate hosts, the commonly used detection method is the post mortem examination of meat in the slaughter houses to check for the presence of hydatid cyst. ELISAs method, use of US and serum antibody detection methods are also being used (4, 12). In dogs, capro-antigen tests are used to detect infections. Microscopical examination and Polymerase Chain reaction (PCR) are used for *E. granulosus* spp. identification (15, 25, 26).

The commonly used interventional method in Kenya is surgery in hospitals. Puncture-aspiration-injection-reaspiration (PAIR) method is being used exclusively in Turkana County and has not been introduced in hospitals. Chemotherapy with albendazole is used especially in inoperable cases or during surgical intervention in hospitals (25). Oxfendazole has been used in sheep and goats and is also suggested to be effective to *E. granulosus* adult in the intestinal tract of dogs (26, 27).

The active control exercises have been spearheaded by AMREF from 1983 to 2012, to control CE in Turkana region and entire Kenya leading to the decline of CE prevalence in Turkana from 7.5% in the 1980s to 3.8% in 2012 (4, 11)

### The economic impact of Cystic Echinococcosis

Approximately USD 20,000 monetary veterinary loses occurs annually in Kenya due to CE. The annual surgery at KMH is about 100 cases and the individual surgery cost is USD 600. Approximately USD 60,000 is used to treat CE patients annually. The indirect economic losses associated with CE have also been reported where affected people cannot perform their duties as a result of the severity of the CE disease or death of their loved ones. The global annual cost of the disease is estimated to be around USD 3 Billion (3, 4).

## Conclusion

CE prevalence in endemic areas of Kenya is still of public health and veterinary concern with predisposing factors still in place. The control methods initiated by AMREF have been very instrumental towards the reduction of CE prevalence in Turkana area. These control methods should be extended to other parts of the country where the CE risk factors are evident. The control methods for the disease should also be done in the wildlife settings where the parasite and the disease reservoirs exist.

Country-wide surveillance of CE cases should be done to generate the information for the policymakers who will undertake the interventional measures. Keen attention should be put to adjourn CE life cycle. Home slaughter of animals for various events should be carefully monitored to ensure that infected offal is not accessible to dogs and other definitive hosts. Proper and free mass screening in prevalent areas and effective treatment of infected individuals should be done. This may reduce the prevalence among the human intermediate host. The government of Kenya should make the funds available to facilitate CE related surveillance concerning. Kenya should emulate methods used by countries like Iceland, Tasmania, New Zealand, Cyprus, Falkland Islands successfully reduced CE in humans and significantly lessened the infections in sheep and dogs. The methods used by these countries include: education, destruction of infected offal by abattoirs and dog de-worming and vigorous follow-ups
